# Case Report: Taxifolin for neurosurgery-associated early-onset cerebral amyloid angiopathy

**DOI:** 10.3389/fneur.2024.1360705

**Published:** 2024-03-19

**Authors:** Maxwell C. Y. Choi, Tiffany H. P. Law, Sirong Chen, William S. K. Cheung, Carmen Yim, Oliver K. S. Ng, Lisa W. C. Au, Vincent C. T. Mok, Peter Y. M. Woo

**Affiliations:** ^1^Department of Neurosurgery, Kwong Wah Hospital, Kowloon, Hong Kong SAR, China; ^2^Research Department, Hong Kong Sanatorium and Hospital, Hong Kong, Hong Kong SAR, China; ^3^Department of Nuclear Medicine and PET, Hong Kong Sanatorium and Hospital, Hong Kong, Hong Kong SAR, China; ^4^Department of Anatomical and Cellular Pathology, Kwong Wah Hospital, Kowloon, Hong Kong SAR, China; ^5^Department of Medicine and Therapeutics, Prince of Wales Hospital, The Chinese University of Hong Kong, Shatin, Hong Kong SAR, China

**Keywords:** early-onset cerebral amyloid angiopathy, intracerebral hemorrhage, amyloid-beta, ^11^C-Pittsburgh compound B positron emission tomography, taxifolin

## Abstract

Cases of iatrogenic cerebral amyloid angiopathy (CAA) have been increasingly reported recently, particularly those associated with neurosurgery. Preclinical studies have shown taxifolin to be promising for treating CAA. We describe a young 42-year-old man with a history of childhood traumatic brain injury that required a craniotomy for hematoma evacuation. He later presented with recurrent lobar intracerebral hemorrhage (ICH) decades later, which was histologically confirmed to be CAA. Serial ^11^C-Pittsburgh compound B positron emission tomography (^11^C-PiB-PET) imaging showed a 24% decrease in global standardized uptake value ratio (SUVR) at 10 months after taxifolin use. During this period, the patient experienced clinical improvement with improved consciousness and reduced recurrent ICH frequency, which may be partly attributable to the potential amyloid-β (Aβ) clearing the effect of taxifolin. However, this effect seemed to have diminished at 15 months, CAA should be considered in young patients presenting with recurrent lobar ICH with a history of childhood neurosurgery, and serial ^11^C-PiB-PET scans warrant further validation as a strategy for monitoring treatment response in CAA for candidate Aβ-clearing therapeutic agents such as taxifolin.

## Introduction

Although experimental seeding of amyloid-β (Aβ) has been demonstrated in murine and primate models (1), the possibility of human Aβ transmission secondary to neurosurgical intervention resulting in iatrogenic cerebral amyloid angiopathy (CAA) has only recently been recognized (2–5). Efforts to elucidate neurosurgical CAA have been made in recent years, as more cases are being reported (6), with a history of cadaveric dural grafts being the major culprit of postulated Aβ deposition of a prion-like nature, akin to iatrogenic Creutzfeldt–Jakob disease (iCJD) (7). Yet, there are gaps in our current understanding of whether possible Aβ transmission in neurosurgery-associated CAA is the underlying pathophysiological process. This uncertainty in pathophysiology extends further to treatment modalities in CAA, as there are currently no effective treatments for curing or halting CAA progression, with Aβ clearance remaining only as a potential therapeutic approach in CAA (8).

Taxifolin is a plant flavonoid that has been widely used as a health supplement for its anti-inflammatory and antioxidant properties (9), with increasing evidence in murine models suggesting that it could be efficacious in treating CAA by inhibiting Aβ fibril formation and promoting Aβ clearance (9–14). However, there are no ongoing clinical trials investigating the use of taxifolin for CAA. This may be in part due to the absence of consensus regarding clinically meaningful biomarkers to monitor CAA treatment response (15). The ^11^C-Pittsburgh compound B (^11^C-PiB) is a positron emission tomography (PET) ligand that binds to Aβ in extracellular plaques and vessel walls, with multiple studies demonstrating the role of ^11^C-PiB-PET as an emerging CAA neuroimaging biomarker (16–18).

**Figure 1:**
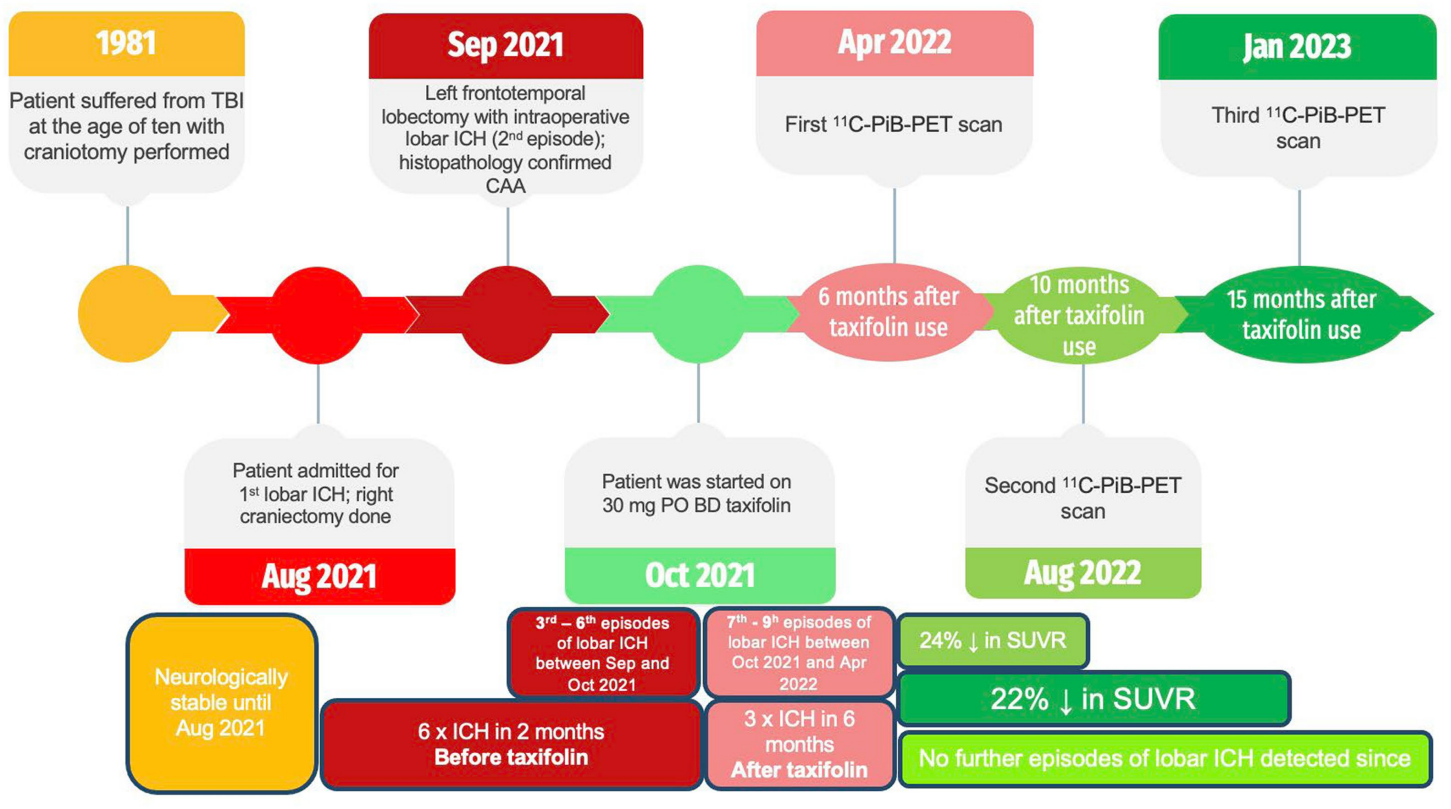
Timeline of important clinical events.

We describe a rare case of a young 42-year-old man with histopathologically confirmed CAA presenting with recurrent lobar intracerebral hemorrhage (ICH) four decades after a previous craniotomy for traumatic brain injury (TBI) and subsequent clinical response to taxifolin. A full timeline of these events is depicted (Figure 1). There is emerging evidence to suggest that this condition is related to Aβ seeding that occurred during the previous open brain surgery decades before (2–6). We also hypothesized that the patient’s subsequent clinical improvement, coupled with radiological evidence of decreased ^11^C-PIB uptake, could be partially attributable to taxifolin use.

Comprehensive multimodal investigations were performed, including neurological examination, MRI (including T2-weighted, FLAIR-weighted, and susceptibility-weighted imaging sequences on a 1.5 T scanner), ^11^C-PiB-PET to assess Aβ deposition, genetic testing for variants associated with hereditary CAA and familial Alzheimer’s disease (AD) via next-generation sequencing (NGS), and histopathological review of brain tissue.

Informed consent from the patient’s next of kin was obtained. This study was approved by the Kowloon Cluster Research Ethics Committee of the Hospital Authority, Hong Kong, and conforms to the Declaration of Helsinki. This study was reported according to the CARE guidelines.

## Case description

We present a young 42-year-old man with a history of severe TBI at the age of 10, sustained from a fall off a playground slide, that resulted in a left acute subdural hematoma. This necessitated a craniotomy for clot evacuation, with no known use of cadaveric dural grafts. The patient enjoyed good health since and was working as a firefighter prior to admission. There was no family history of cerebrovascular or neurodegenerative diseases such as CAA or AD.

**Figure 2:**
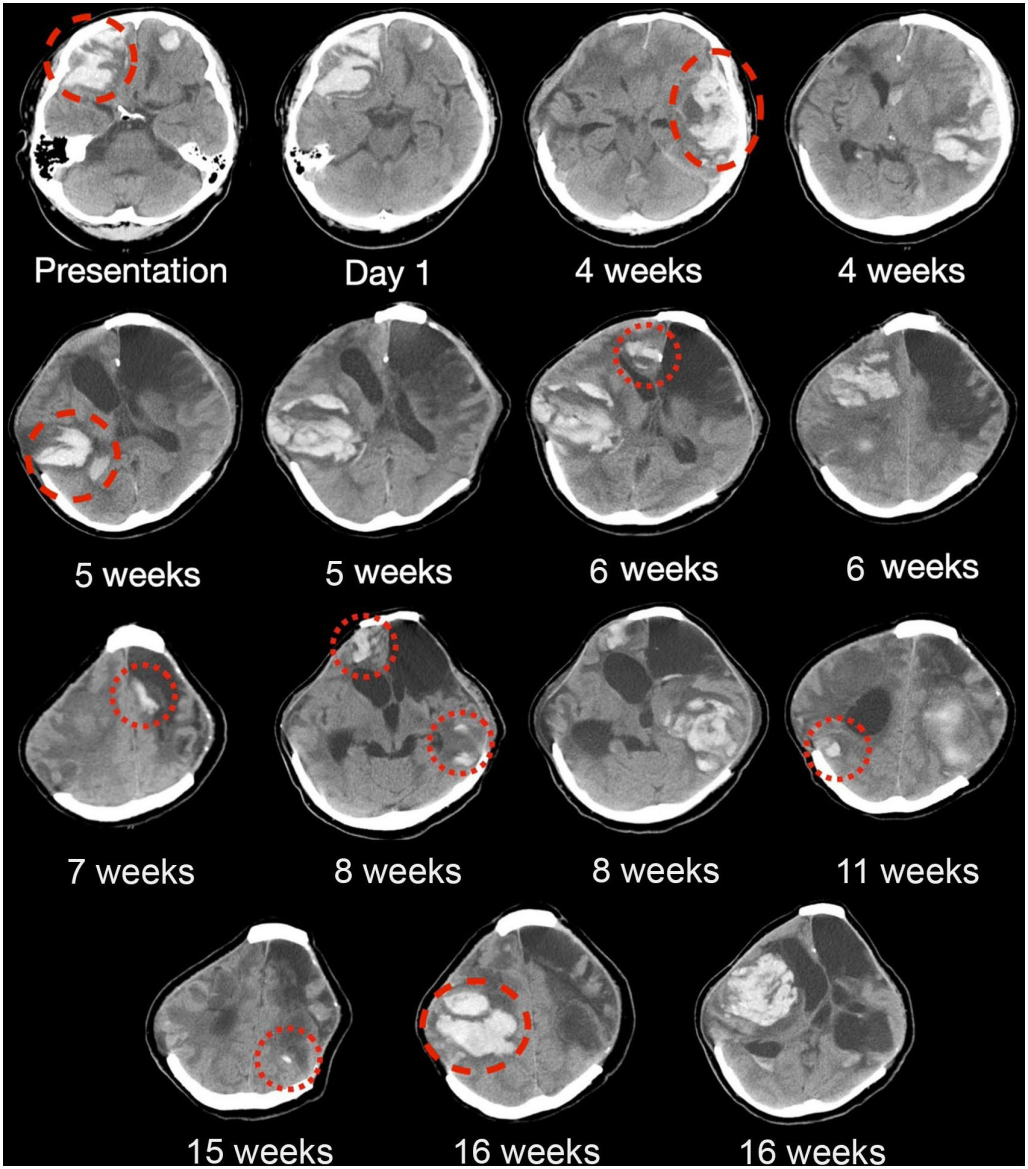
Serial CT brain scans in the first 6 months of presentation. Serial brain computed tomography (CT) findings of a patient with histopathologically confirmed, neurosurgery-associated iatrogenic cerebral amyloid angiopathy (CAA) showing recurrent lobar intracranial hemorrhage (red circles) in the first 6 months since admission. Taxifolin was commenced after around 8 weeks, as shown in the figure, with six new episodes of lobar ICH before taxifolin use and only three new episodes of lobar ICH after taxifolin use, with no newly detected lobar ICH since.

Four decades later, he experienced a spontaneous headache with right upper limb focal seizures while swimming. On admission, he was fully conscious with no focal neurological deficit. A computed tomography (CT) and magnetic resonance imaging (MRI) scan revealed bilateral frontal lobar ICH with significant mass effect (Figure 2; Supplementary Figure S1). CT angiography and magnetic resonance venography did not reveal an underlying vascular lesion or dural venous sinus thrombosis. Investigations for blood coagulopathy, thrombophilia, and vasculitis markers were also unremarkable.

The patient experienced neurological deterioration soon after admission when the Glasgow Coma Score (GCS) dropped to 13/15 (E3V4M6) with anisocoria and left hemiplegia. A repeat scan showed expansion of the right frontal lobar ICH that required an emergency right decompressive craniectomy. Serial scans showed gradual resolution of the residual ICH, and the patient recovered full consciousness (Figure 2). Four weeks following the first surgery, the patient experienced a second episode of acute deterioration, with GCS dropping to 10/15 (E3V2M5) and right hemiplegia.

**Figure 3:**
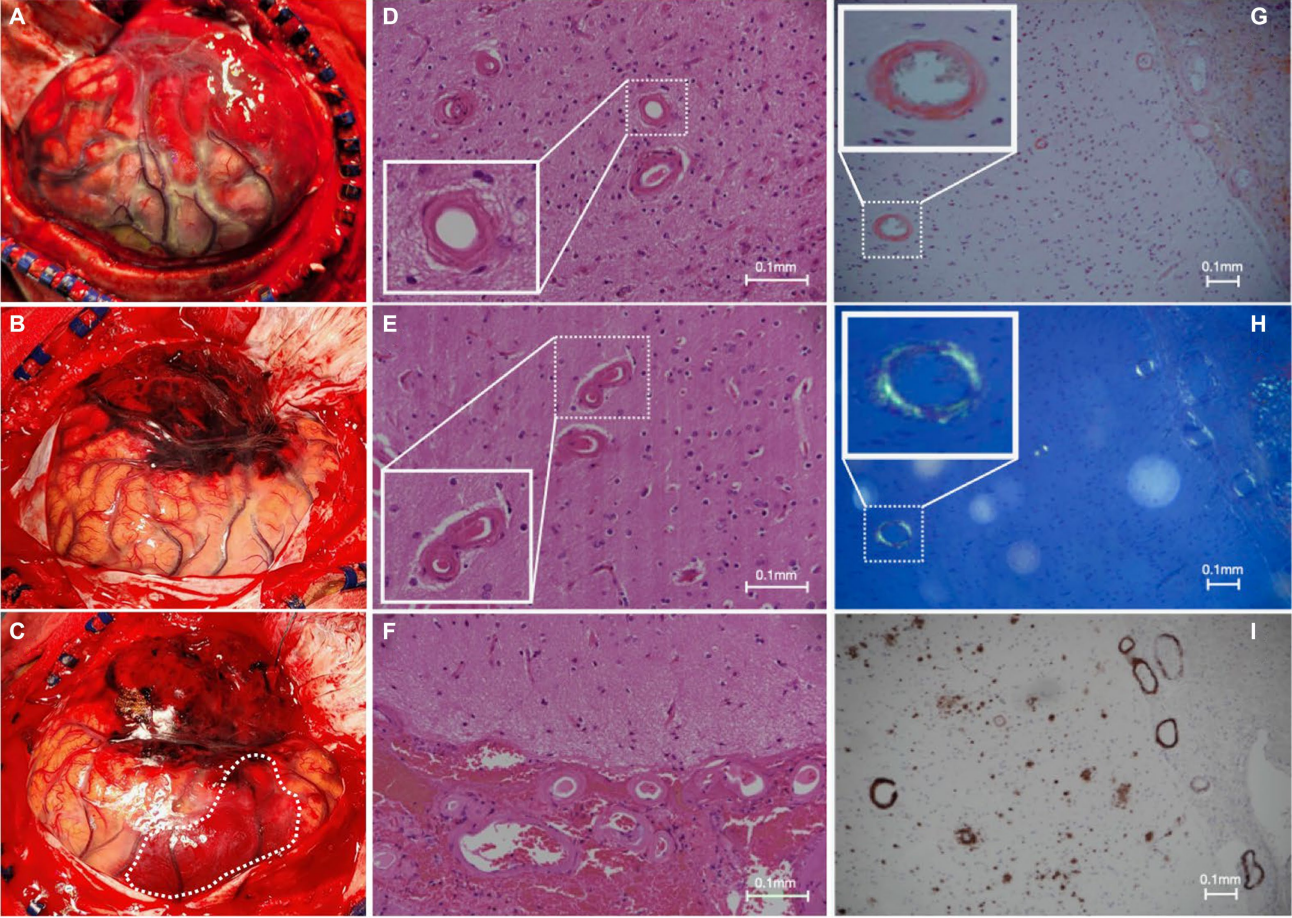
Intraoperative and histopathological findings. **(A)** Intraoperative view of grossly swollen brain parenchyma with significant rebleeding in the left anterior temporal lobe; and **(B,C)** frontal subcortical intracerebral hemorrhage (white dotted line) during a left-sided decompressive craniectomy in which left anterior frontotemporal lobectomy was performed; **(D–F)** hematoxylin and eosin slides of the specimen demonstrate extensive replacement of the arteriolar smooth muscle layer with extracellular eosinophilic material; **(G)** Congo red staining shows salmon-pink appearing vessels; **(H)** with apple-green birefringence under polarized light; and **(I)** positive immunohistochemistry for involvement of Aβ.

A new contralateral left temporal lobar ICH was detected on CT, and a left decompressive craniectomy was performed. Intraoperatively, spontaneous rebleeding of the anterior temporal lobe and a frontal lobar ICH were observed that required further clot evacuation (Figures 3A–C). Histopathological examination of the resected brain tissue confirmed the diagnosis of CAA (Figures 3D–I). A targeted NGS panel of 11 genes associated with hereditary cerebral small vessel disease (*APP, PSEN1, PSEN2, CST3, IMT2B, CBS, COL4A1, COL4A2, FOXC1, GLA, HTRA1, NOTCH3,* and *TREX1*) did not show any pathogenic mutations. APOE genotyping revealed an e3/e4 genotype, but there was no clinical evidence of familial CAA. The patient remained comatose with a GCS of 7/15 and experienced recurrent episodes of lobar ICH at multiple sites. Taxifolin (100 mg per tablet; 300 mg BD) was prescribed 8 weeks after admission following the sixth episode of lobar ICH. The patient was successfully weaned off mechanical ventilation 6 months after starting taxifolin and attained a minimally conscious state. No further episodes of ICH have been noted since. No taxifolin-associated adverse effects were observed.

**Figure 4:**
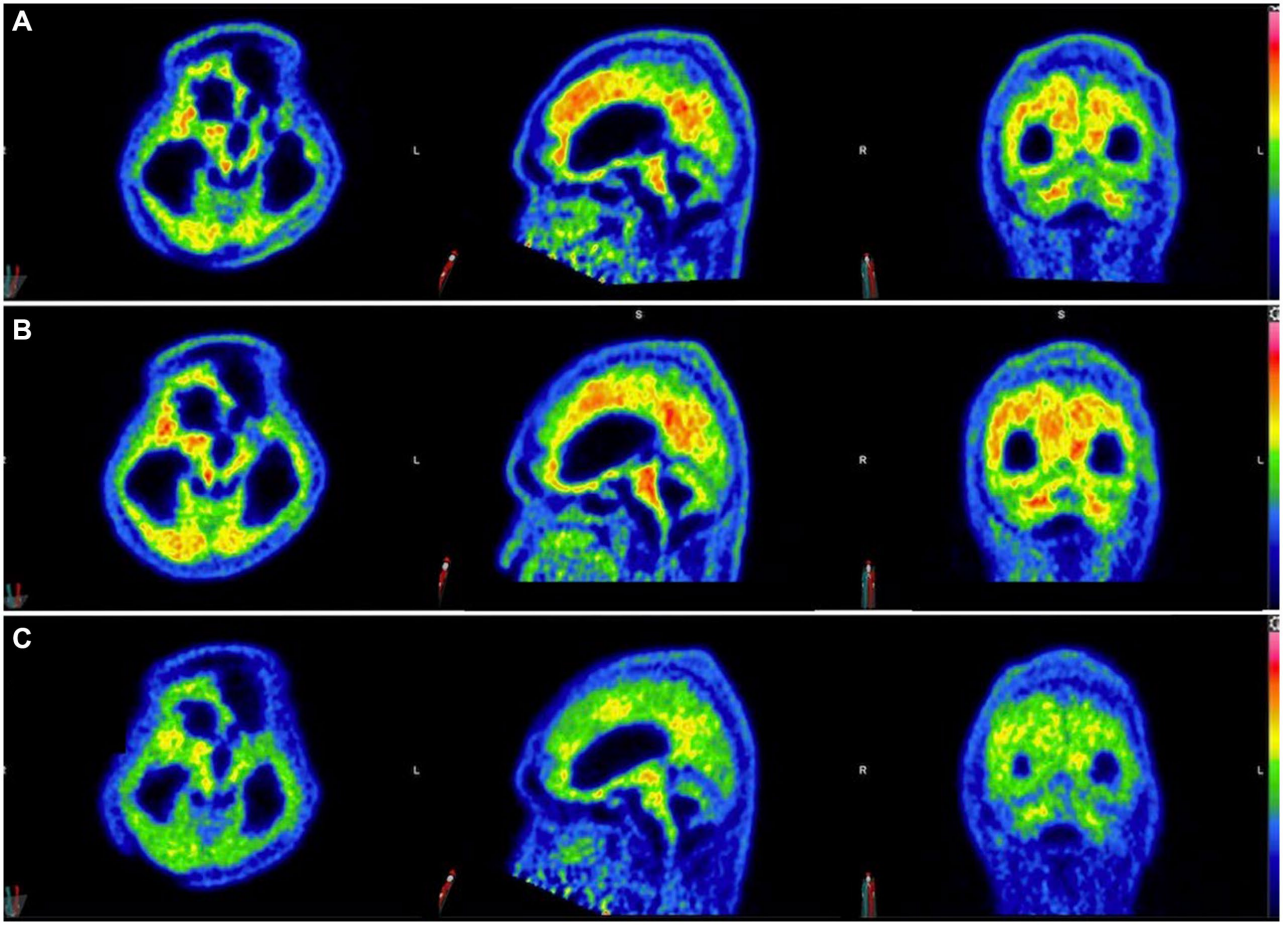
Serial ^11^C-Pittsburgh compound B positive emission tomography (^11^C-PiB-PET) scans following taxifolin use. Serial ^11^C-PiB-PET scans in axial, sagittal, and coronal views followed in response to taxifolin use after 6 months **(A)**, 10 months **(B)**, and 15 months **(C)**, respectively. Visually, amyloid deposition can be gaged according to the colored bar on the right, which corresponds to the cortical-to-cerebellar standardized uptake volume ratio (SUVR; red = high uptake, yellow-green = moderate uptake, blue = least uptake). Note that the semi-quantification parameter cortical-to-cerebellum SUVR is equal to “SUV_mean_/SUV_mean_ in cerebellum,” where SUV_mean_ is the mean SUV within each cortical volume of interest.

Three ^11^C-PiB-PET scans were performed at 5-month intervals starting after 6 months of taxifolin administration after the patient was stabilized (Figure 4). Serial scans were arranged to quantify changes in Aβ deposition at 13 regions of interest by determining the cortical-to-cerebellum standardized uptake value ratio of ^11^C-PiB (SUVR)—in particular, the second ^11^C-PiB-PET scan revealed a 24% decrease in global Aβ deposition compared to the index ^11^C-PiB-PET scan, whereas the third scan demonstrated a comparable 22% decrease compared to the index ^11^C-PiB-PET (Supplementary Table S1). In the first 6 to 10 months after taxifolin administration, a significant decrease in Aβ deposition was noted, as quantified by a 2–77% decrease in the SUVR across all the cortical regions of interest (Supplementary Table S1; Supplementary Figure S2). During this period, the patient experienced clinical improvement in terms of the ability to wean off mechanical ventilation and improved consciousness. He currently requires ongoing neurorehabilitation.

## Discussion

We describe a rare case of early-onset CAA presenting with recurrent lobar ICH. We speculated that this could be associated with previous open brain surgery for TBI nearly four decades prior. The latency period between probable initial Aβ exposure and CAA onset for our patient was 36 years, which is consistent with an average latency period of 34 ± 5 years as reported in the literature (5). A systematic review of 23 patients with early-onset iatrogenic CAA diagnosed between 2012 and 2022 had a mean age of first presentation of 37.7 ± 8.1 years (5). This is in contrast to sporadic CAA, which is seldom reported before the sixth decade of life (5). Furthermore, all of the previous cases (23/23; 100%) had a history of childhood neurosurgery, yet only 35% (10/23) could be attributed to cadaveric dural grafts as a source of exposure. Therefore, neurosurgical CAA, with or without cadaveric dural graft involvement, is increasingly being recognized, but in the absence of direct evidence, the pathogenesis remains speculative.

Several hypotheses could explain the decades-long latency. First, human cadaveric dural grafts may have been the source of Aβ protein seeding. Reports of direct Aβ proteopathic seed inoculation, akin to prion diseases, have been well-described in several animal studies and in sporadic patient case reports (2, 3). However, we confirm that no such dural graft was utilized (Figures 3A–C). Another possible source of Aβ exposure could have been contamination from surgical instruments, which is an established mode of transmission for prion diseases such as iCJD (7). Therefore, it is believed that Aβ seeding via surgical instrumentation was the most likely mode of transmission for our patient.

Currently, there is no known effective disease-modifying therapeutic agent to treat CAA. Taxifolin has been well validated in murine models as a potential therapeutic agent for its role in the inhibition of Aβ fibril formation and Aβ disassembly (9–14), Therefore, we decided to use taxifolin only on the grounds of compassionate treatment, as the recurrent lobar ICH was refractory to surgical intervention. The dosing regimen of taxifolin used was determined based on the typical dose used as a dietary supplement. We also present the first attempt at performing radiological semi-quantification of Aβ deposition by utilizing serial ^11^C-PiB-PET scans. The significant reduction in SUVRs was most pronounced in the cerebellar vermis, mesial temporal lobes, frontal gyri, and thalami (Supplementary Table S1; Supplementary Figure S1). Given that these four regions are known to be involved in executive function, attention, and memory, we postulate that this may explain the partial neurological recovery of the patient, possibly in regaining consciousness (19). It is unknown why there was no further reduction in Aβ deposition beyond 10 months, but it may be because taxifolin dosage was not adjusted, and a plateau effect was established by this timepoint.

This study has several limitations. Due to resource, cost, and manpower limitations at our institution, serial MRI scans beyond the index scan were not available to monitor CAA progression, if any. Moreover, since the patient had been ventilator-dependent for 8 months, ^11^C-PiB-PET scanning to establish a baseline in Aβ burden before taxifolin use could not be safely performed. There has been one report of decreased amyloid burden on serial ^11^C-PiB-PET scans in a case of CAA-related inflammation (CAA-ri), which may suggest post-inflammatory amyloid clearance (18); however, there was no imaging or histopathological evidence in our patient to demonstrate CAA-ri. Recurrent lobar ICH may also potentially distort baseline SUVR calculations, but since no further lobar ICH was noted following taxifolin use, comparing percentage changes in the SUVR relative to the first scan was regarded as a reasonable estimate. Furthermore, the clinical improvement of the patient could also be explained by gradual hematoma resorption along with taxifolin use. Finally, the long-term effects of taxifolin administration have yet to be determined, but after 24 months of use, no adverse effects were identified. Further studies are required to verify the role of ^11^C-PiB-PET in monitoring CAA progression and as a potential tool for assessing treatment responses for candidate therapeutic agents such as taxifolin.

In summary, we describe a rare case of early-onset neurosurgical CAA treated with taxifolin. It was speculated that Aβ transmission occurred during neurosurgery decades before CAA-associated lobar ICH. This has major implications not only in terms of clinical management but also raises concerns about a possible novel prion-like transmission of Aβ in humans by neurosurgical inoculation. Our findings suggest that serial ^11^C-PiB-PET scans may be a clinically useful neuroimaging biomarker to monitor CAA progression, and the efficacy of taxifolin as a potential therapeutic agent for CAA needs to be confirmed with prospective clinical trials.

## Data availability statement

The datasets presented in this article are not readily available because of ethical and privacy restrictions. Requests to access the datasets should be directed to the corresponding author.

## Ethics statement

The studies involving humans were approved by the Kowloon Cluster Research Ethics Committee of the Hospital Authority, Hong Kong. The studies were conducted in accordance with the local legislation and institutional requirements. The participants provided their written informed consent to participate in this study. Written informed consent was obtained from the individual(s) for the publication of any potentially identifiable images or data included in this article.

## Author contributions

MC: Conceptualization, Data curation, Formal analysis, Investigation, Methodology, Project administration, Software, Validation, Visualization, Writing – original draft, Writing – review & editing. TL: Data curation, Investigation, Methodology, Project administration, Resources, Writing – original draft, Writing – review & editing. SC: Data curation, Formal analysis, Investigation, Software, Validation, Writing – original draft, Writing – review & editing. WC: Data curation, Investigation, Project administration, Software, Validation, Writing – original draft, Writing – review & editing. CY: Data curation, Investigation, Methodology, Writing – original draft, Writing – review & editing. ON: Data curation, Formal analysis, Investigation, Methodology, Writing – original draft, Writing – review & editing. LA: Data curation, Formal analysis, Investigation, Project administration, Resources, Validation, Writing – original draft, Writing – review & editing. VM: Data curation, Investigation, Methodology, Project administration, Resources, Supervision, Validation, Writing – original draft, Writing – review & editing. PW: Conceptualization, Data curation, Formal analysis, Investigation, Methodology, Project administration, Resources, Resources, Supervision, Validation, Visualization, Writing – original draft, Writing – review & editing.

## References

[1] Lam S Petit F Hérard A-S Boluda S Eddarkaoui S Guillermier M. Transmission of amyloid-beta and tau pathologies is associated with cognitive impairments in a primate. Acta Neuropathol Commun. (2021) 9:165. doi: 10.1186/s40478-021-01266-8, PMID: 34641980 PMC8507137

[2] Banerjee G Adams ME Jaunmuktane Z Alistair Lammie G Turner B Wani M. Early onset cerebral amyloid angiopathy following childhood exposure to cadaveric dura. Ann Neurol. (2019) 85:284–90. doi: 10.1002/ana.25407, PMID: 30597599 PMC6492172

[3] Giaccone G Maderna E Marucci G Catania M Erbetta A Chiapparini L. Iatrogenic early onset cerebral amyloid angiopathy 30 years after cerebral trauma with neurosurgery: vascular amyloid deposits are made up of both AΒ40 and AΒ42. Acta Neuropathol Commun. (2019) 7:70. doi: 10.1186/s40478-019-0719-1, PMID: 31046829 PMC6498603

[4] Jaunmuktane Z Quaegebeur A Taipa R Viana-Baptista M Barbosa R Koriath C. Evidence of amyloid-β cerebral amyloid angiopathy transmission through neurosurgery. Acta Neuropathol. (2018) 135:671–9. doi: 10.1007/s00401-018-1822-2, PMID: 29450646 PMC5904220

[5] Banerjee G Samra K Adams ME Jaunmuktane Z Parry-Jones AR Grieve J. Iatrogenic cerebral amyloid angiopathy: an emerging clinical phenomenon. J Neurol Neurosurg Psychiatry. (2022) 93:693–700. doi: 10.1136/jnnp-2022-328792, PMID: 35577510

[6] Kaushik K van Etten ES Siegerink B Kappelle LJ Lemstra AW Schreuder FHBM. Iatrogenic cerebral amyloid Angiopathy post neurosurgery: frequency, clinical profile, radiological features, and outcome. Stroke. (2023) 54:1214–23. doi: 10.1161/STROKEAHA.122.041690c, PMID: 37035916 PMC10121246

[7] Cali I Cohen ML Haїk S Parchi P Giaccone G Collins SJ. Iatrogenic Creutzfeldt-Jakob disease with amyloid-β pathology: an international study. Acta Neuropathol Commun. (2018) 6:5. doi: 10.1186/s40478-017-0503-z, PMID: 29310723 PMC5759292

[8] Kozberg MG Perosa V Gurol ME van Veluw SJ. A practical approach to the management of cerebral amyloid angiopathy. Int J Stroke. (2021) 16:356–69. doi: 10.1177/1747493020974464, PMID: 33252026 PMC9097498

[9] Liu Y Shi X Tian Y Zhai S Liu Y Xiong Z. An insight into novel therapeutic potentials of Taxifolin. Front Pharmacol. (2023) 14:855. doi: 10.3389/fphar.2023.1173855 PMC10227600 37261284

[10] Saito S Tanaka M Satoh-Asahara N Carare RO Ihara M. Taxifolin: a potential therapeutic agent for cerebral amyloid angiopathy. Front Pharmacol. (2021) 12:643357. doi: 10.3389/fphar.2021.643357 33643053 PMC7907591

[11] Tanaka M Saito S Inoue T Satoh-Asahara N Ihara M. Potential therapeutic approaches for cerebral amyloid angiopathy and alzheimer’s disease. Int J Mol Sci. (2020) 21:1992. doi: 10.3390/ijms21061992, PMID: 32183348 PMC7139812

[12] Tanaka M Saito S Inoue T Satoh-Asahara N Ihara M. Novel therapeutic potentials of Taxifolin for amyloid-β-associated neurodegenerative diseases and other diseases: recent advances and future perspectives. Int J Mol Sci. (2019) 20:2139. doi: 10.3390/ijms20092139, PMID: 31052203 PMC6539020

[13] Inoue T Saito S Tanaka M Yamakage H Kusakabe T Shimatsu A. Pleiotropic neuroprotective effects of Taxifolin in cerebral amyloid angiopathy. Proc Natl Acad Sci. (2019) 116:10031–8. doi: 10.1073/pnas.1901659116, PMID: 31036637 PMC6525485

[14] Saito S Yamamoto Y Maki T Hattori Y Ito H Mizuno K. Taxifolin inhibits amyloid-β oligomer formation and fully restores vascular integrity and memory in cerebral amyloid angiopathy. Acta Neuropathol Commun. (2017) 5:26. doi: 10.1186/s40478-017-0429-5, PMID: 28376923 PMC5379578

[15] Greenberg SM Salman RA-S Biessels GJ van Buchem M Cordonnier C Lee JM. Outcome markers for clinical trials in cerebral amyloid angiopathy. Lancet Neurol. (2014) 13:419–28. doi: 10.1016/S1474-4422(14)70003-1, PMID: 24581702 PMC4085787

[16] Chang Y Liu J Wang L Li X Wang Z Lin M. Diagnostic utility of integrated 11C-Pittsburgh compound B positron emission tomography/magnetic resonance for cerebral amyloid angiopathy: a pilot study. Frontiers in aging. Neuroscience. (2021) 13:721780. doi: 10.3389/fnagi.2021.721780, PMID: 34899265 PMC8660657

[17] Farid K Charidimou A Baron JC. Amyloid positron emission tomography in sporadic cerebral amyloid angiopathy: a systematic critical update. Neuroimage Clin. (2017) 15:247–63. doi: 10.1016/j.nicl.2017.05.002, PMID: 28560150 PMC5435601

[18] Yang JY Chu YT Tsai HH Jeng JS. Amyloid and tau PET in cerebral amyloid angiopathy-related inflammation two case reports and literature review. Front Neurol. (2023) 14:1153305. doi: 10.3389/fneur.2023.1153305, PMID: 37188315 PMC10175602

[19] D’Angelo E Casali S. Seeking a unified framework for cerebellar function and dysfunction: from circuit operations to cognition. Front Neural Circuits. (2013) 6:116. doi: 10.3389/fncir.2012.00116, PMID: 23335884 PMC3541516

